# **Self-Burns in Fars Province, Southern Iran**

**Published:** 2016-01

**Authors:** Ali Akbar Mohammadi, Hamid Reza Tohidinik, Mitra Zardosht, Seyed Morteza Seyed Jafari

**Affiliations:** 1 Shiraz Burn Research Center, Division of Plastic and Reconstructive Surgery, Department of Surgery, Shiraz University of Medical Sciences, Shiraz, Iran;; 2 School of Public Health, Gonabad University of Medical Sciences, Gonabad, Iran

**Keywords:** Incidence, Self-burn, Trend, Iran

## Abstract

**BACKGROUND:**

The alarming incidence of self- burning provoked to set up a multidisciplinary preventive program to decrease the incidence and complications of this harmful issue. This study investigated the incidence and the preventive measures in self-burn in Fars Province, southern Iran.

**METHODS:**

This study was a longitudinal prospective design on trend of self-inflicted burn injuries in Fars province after setting up a regional multidisciplinary preventive plan (2009-2012).

**RESULTS:**

From 18862 admitted patients, 388 (2%) committed self-burning. While the incidence showed a constant decrease in proportion of suicidal cases among all admitted patients (2.5% to 1.6%). The mean age of self-burning victims ranged from 28.3±10.8 to 30.3±11.7 years. The female victims comprised 67.4% of all suicidal burn patients (Female to male ratio: 2.18). The leading causes of suicide commitment were familial conflicts (75.6%) and psychological problems (16.7%)

**CONCLUSION:**

It is crucial to continue the regional preventive programs and pave the way to set up national, and even international collaborations to alleviate relevant financial, social, cultural and infrastructural difficulties in order to have lower incidence for this dramatic issue.

## INTRODUCTION

Suicide is an awful way to die by a person in the full knowledge or expectation of its outcome. The incidence, methods and means in suicide vary according to geographical region, social factors, cultures and gender.^1^ Self-burning is the most devastating forms of suicide attempt, while, it is not only a life-threatening problem for the injured patient but also the survivors and their societies should deal with serious physical, emotional, mental, psychological, and financial problems.^2^^,^^3^

Burn is still considered devastating in emergency medicine leading to physical and psychological disabilities^1^ and has an increasing mortality and morbidity for mother and child during pregnancy.^2^ For all survivors from burn, one of the most problems is scarring having psychological effects for the burn patient.^3^ There may be resistance of several bacteria in burn injuries to common therapies complicating the situation more such as administered silver sulfadiazine,^4^ even there have been efforts for treatment of burn wounds with medications having less adverse effects and more efficacy, such as medicinal herbals^5^^-^^9^ while still they cannot reduce the psychological stresses of burn injuries. 

Various factors, including imitation of others’ symbolic acts, political protest, and ritual suicide that can be considered as the reasons for self-burning.^10^ These injuries are commonly associated with previous psychiatric disorders or predisposing factors such as: alcohol, substance abuse, relationship discords, unemployment and emotional trauma.^11^^,^^12^

Self-immolation is uncommon in high-income countries (Western Europe and the United States) and accounts for 1 to 2% of suicides.^13^^,^^14^ However, it is more frequent in low-income and middle-income countries, especially in the Middle East, Africa, and South Asia.^11^^,^^15^^,^^16^ Self-immolation has been reported to account for as much as 40% to 71% of suicides in some areas of the developing world.^10^^,^^17^^,^^18^ In different parts of Iran, 1.39 to 40.3% of all suicides and para-suicides have been attributed to self-inflicted burns.^19^

This alarming incidence of self- burning in Iran and especially in Ghotbeddin Shirazi Burn Hospital, the burn referral center for southwest of Iran, provoked us to set up a multidisciplinary program to determine the incidence and complications of this harmful issue. In this study we investigated the incidence, characteristics, outcome, and trend of self-inflicted burn injuries in Shiraz Burn Hospital, one of the biggest burn centers in Iran between 2009 and 2012.

## MATERIALS AND METHODS

The province of Fars is located in the South west of Iran. The population is 4,596,658, living in 122,608 km^2^ while 67.6% of the population reside in rural areas and the rest are urban residents. Ghotbeddin Shirazi Hospital is the only burn center in this area, affiliated to Shiraz University of Medical Sciences. Because of its designation as the burn referral center for the entire province, all significant burn cases in Fars province are referred to this burn unit. 

This study was performed in a longitudinal prospective design on trend of self-inflicted burn injuries in Fars province after setting up a multidisciplinary plan, due to alarming incidence of self-burning attempts in Fars Province.^11^^,^^17^ All self-burned patients admitted to Ghotbeddin Shirazi Hospital from 2009 to 2012 were evaluated. Data collected included age, gender, employment, educational level, percentage of burn in terms of body surface, etiology, method, and the outcome of treatment. The total burned body surface area (TBSA) was estimated from body surface charts. 

Self-inflicted burn was suspected on the patient’s or a reliable witness’ confession to deliberate self-burning, or the physician’s suspicion during time of admission or hospital stay. In addition, if available, the records of the police or emergency services were taken into consideration. Self-inflicted burn was confirmed by the psychiatrist. Additional interviews were carried out with parents, spouse, siblings, children, or friends who accompanied the patients and knew them for a long time or had frequent contact with them to obtain the fullest accounts. 

In the period of the study, the actual treatment and management protocol of burns at this center was generally followed by conventional methods including daily washing, debridement, and topical antibiotics for burns until they healed primarily or granulation tissue appeared. Thereafter, delayed skin grafting was done for full thickness skin loss. All analyses were conducted using the Statistics Package for the Social Sciences (SPSS; SPSS Inc., Chicago, IL, USA, version 21.0). Data were expressed as mean±SD.

## RESULTS

From 18862 admitted patients to Ghotbeddin Shirazi Hospital, 388 (2.0%) committed self-burning. While, incidence of this event, showed a constant decrease in proportion of suicidal cases among all admitted patients (from 2.5% to 1.6%) during this time period (Table 1). The mean age of self-burning victims ranged from 28.3±10.8 to 30.3±11.7 years (Table 2), and their mean total body surface area (TBSA) was between 52.9±30.0 and 65.8±36.7 during this time period. 

**Table 1:** Trend of different kinds of burning between 2009 and 2012 in Fars province, Iran

**No. (%)**	**2009**	**2010**	**2011**	**2012**	**Total**
Accidental	4598 (97.3)	4407 (96.8)	4850(98.0)	4553 (98.2)	18408 (97.6)
Homicidal	12 (0.3)	33 (0.7)	15 (0.3)	6 (0.1)	66 (0.4)
Suicidal	116 (2.5)	112 (2.5)	84 (1.7)	76 (1.6)	388 (2.0)
Total	4726 (100.0)	4552 (100.0)	4949 (100.0)	4635 (100.0)	18862 (100.0)

**Table 2:** Age of burn in self-burning patients during 2009-2012

		**2009**	**2010**	**2011**	**2012**
Age	Mean (SD)	28.3 (10.8)	29 (10.3)	30.3 (11.7)	29.6 (12.4)
	Median (min-max)	26 (7-83)	27 (9-70)	28 (13-75)	26 (15-77)

Evaluation of self-burning victims showed that female victims comprised 67.4% (ranged from 56.6% to 76%) of all suicidal burn patients in the period of study (Female: male ratio 2.18). This proportion was gradually getting closer to male victims and these suicidal attempts occurred more (57.7%) in rural areas (ranged from 52.6% to 62%) while 17.8% of the patients were completely illiterate and around 65% of them could finish their secondary education level, and just 3.2% of them had university degree (Table 3).

**Table 3:** Characteristics of self-burning patients during 2009-2012

**Variable**		**2009 ** **No. (%)**	**2010 ** **No. (%)**	**2011 ** **No. (%)**	**2012 ** **No. (%)**	**Total ** **No. (%)**
Gender	Male	28 (24.1 )	31 (27.7)	30 (35.7)	33 (43.4)	122 (31.4)
	Female	88 (75.9)	81 (72.3)	54 (64.3)	43 (56.6)	266 (68.6)
Living place	Rural	67 (57.8)	65 (58.0)	52 (61.9)	40 (52.6)	224 (57.7)
	Urban	49 (42.2)	47 (42.0)	32 (38.1)	36 (47.4)	164 (42.3)
Hospitalization	Outpatient	7 (6.0)	12 (10.7)	5 (6.0)	1 (1.3)	25 (6.4)
	Hospitalized	109 (94.0)	100 (89.3)	79 (94.0)	75 (98.7)	363 (93.6)
Educational level	Illiterate	20 (17.2)	22 (19.6)	13 (15.5)	14 (18.4)	69 (17.8)
	Under diploma	84 (72.4)	69 (61.6)	56 (66.7)	45 (59.2)	254 (65.5)
	Diploma	11 (9.5)	16 (14.3)	11 (13.1)	13 (17.1)	51 (13.1)
	Graduated	1 (0.9)	5 (4.5)	4 (4.8)	4 (5.3)	14 (3.6)

The leading causes of suicide commitment among patients was familial conflicts (75.6% l), and psychological problems (16.7%) (Table 4). Flame was the most common etiology of burns, and the common mechanisms for self-infliction in our patients were using an accelerant such as petrol and gasoline (Table 5).

**Table 4:** Causes of suicide commitment among patients

**Variable**	**2009 ** **No. (%)**	**2010 ** **No. (%)**	**2011 ** **No. (%)**	**2012 No. (%)**	**Total ** **No. (%)**
Familial conflicts	80 (69.0)	89 (79.5)	65 (77.4)	55 (72.4)	289 (74.5)
Mental health problems	20 (17.2)	18 (16.1)	12 (14.3)	17 (22.4)	67 (17.3)
Financial problems	6 (5.2)	1 (0.9)	1 (1.2)	1 (1.3)	9 (2.3)
Feelings of guilt	3 (2.6)	1 (0.9)	2 (2.1)	3 (3.9)	9 (2.3)
Jobless life	4 (3.4)	0 (0)	1 (1.2)	0 (0)	5 (1.3)
The death of a loved one	1 (0.9)	1 (0.9)	3 (3.6)	0 (0)	5 (1.3)
Academic failure	1 (0.9)	0 (0)	0 (0)	0 (0)	1 (0.3)
Love failure	0 (0)	1 (0.9)	0 (0)	0 (0)	1 (0.3)
Protest	1 (0.9)	1 (0.9)	0 (0)	0 (0)	2 (0.5)
Total	116 (100)	112 (100)	84(100)	76 (100)	388 (100)

**Table 5:** Mechanism for self-infliction

**Variable**	**2009 ** **No. (%)**	**2010 ** **No. (%)**	**2011 ** **No. (%)**	**2012 ** **No. (%)**	**Total ** **No. (%)**
Petroleum	91 (78.4)	77 (68.7)	53 (63.1)	50 (65.8)	271 (69.8)
Gasoline	14 (12.1)	19 (17.0)	16 (19.0)	21 (27.6)	70 (18)
Others	11 (9.5)	16 (14.3)	15 (17.9)	5 (6.6)	47 (12.1)
Total	116 (100)	112 (100)	84 (100)	76 (100)	388 (100)

## Discussion

Previous studies from our center showed alarming incidence of self-burning compared to Western countries.^11^^,^^20^ Therefore, we set up a regional multidisciplinary program against self- burning. The prospective analysis of admitted burn patients in Shiraz Burn Center, as the main burn referral center in southwest of Iran from 2009 to 2012 showed a decrease in incidence of self-burning in all patient groups. 

A variety of conditions can put people at increased risk of self-immolation, such as interpersonal conflicts; cultural, psychosocial, religious, legal problems; poor socioeconomic status.^10^^,^^11^^,^^21^ Prevention programs should aim at modifying these risk factors and need to be executed with patience, persistence, and precision, targeting high-risk groups.^22^ Education, publicity, product design/environmental change, and legislation and regulation, are the main strategies to reduce harm from burn injuries.^11^^,^^22^^,^^23^ Similarly, different factors could be discussed as causes to decrease the incidence and poor outcome of self-burning in our center.

Previous studies in our center showed that suicide attempts by burning accounted for 14.5% and 24.8% of all burn patients were admitted to the hospital.^11^^,^^20^ As a result, the Regional Committee for Injury and Burn Prevention and Control was established in Shiraz Burn Research Center to coordinate all burn prevention programs and collaborate between different parts of the society. This committee set up a multidisciplinary preventive package to reduce the incidence of self- burning attempt in Fars Province. The main topics of this program are listed as follows.

It is important to promote a governmental organization in any effective burn prevention program.^22^ In previous reports from our center, self-inflicted burns were noted mainly with patients of low level of literacy and education.^20^ As a result, selected clergymen, health workers, school health staff, psychologists were informed about the different aspects of suicide and self-burning, in the regular training workshops, seminars, and conferences. Then, they became teacher ambassadors of the preventive programs to make their audiences familiar with incidence, complications, and the preventive strategies of this crucial issue.

Different training packages (Films, pamphlets, articles, DVDs) about preventive strategies against self-burning were prepared; then, distributed among high risk families, and published in public media. In order to attract the attentions of the families and making the students familiar with this deathful issue, painting competitions under topic of preventive strategies in burn trauma were held among the students annually.

In Asia, victims were grossly about 10 years younger than in Europe.^21^ The mean age of self-inflicted burns ranged from 28.3±10.8 to 30.3±11.7 years and their mean percentage of burn was between 52.9±30.0 and 65.8±36.7 during this time period. In a similar study by Ahmadi et al., the majority of patients were younger than 30 years of age.^10^ likewise, the mean age of completed suicide by self-burning in the study conducted by Saadat et al. was 26.7±13.5 years for females and 29.3±9.9 years for males.^24^

Literature review shows that generally men are more likely to commit suicide, while women have more suicide attempts.^24^^-^^26^ In Iran, most men who committed suicide hanged themselves, whereas 83% of women who committed suicide set themselves ablaze.^19^ Female: male ratio in the previous studies from our center was 6.4 and then 2.5, which is more than the present study (2.18).^11^^,^^20^ Ahmadi et al. stated in their studies that women were the main victims of self-burning in Iran. They account for 70–88% of all self-immolation admissions to burn centers in Iran.^10^^,^^16^ Many other studies however, have found a preponderance of women in self-immolation suicide and while others report no sex difference at all.^13^^,^^24^^,^^27^

The higher incidence rate of deliberate self-burning among women may be related to: i) the major changes happening at young ages in woman’s life with no perspective solution of individual or family problems such as addiction of the spouse, the difference of age between the married couple, lack of understanding with the spouse, bigamy, lack of interest in the family affairs, lack of love, premature marriage and excessive sensitivity in regard to the taboo of divorce; ii) low socioeconomic class, unemployment, illiteracy, unequal opportunity for the two sexes, lesser respect for women, and the traditional male domination social codes in the communities, and crowded families; iii) conservative traditions, deprivations and social restrictions, especially in rural communities of Iran, where women have little hope in life and often face a grim future; iv) easy access to inflammable agents, the style of traditional Iranian women’s dress with large surface and volume of clothes and synthetic-type and flammable materials.^10^^,^^11^^,^^19^

Self-immolation is linked to low educational level.^12^ In the previous studies from our center, 82% of self-inflicted burn patients had low level of literacy,^20^ and only 1.7% of the victims had university degrees.^11^ While, in the current study, an average of 17.8% of the patients were completely illiterate and just 3.2% of them had university degree.

Serious emotional problems make people more susceptible to suicidal thoughts and attempts.^13^ Previous psychiatric problems, often depression or borderline personality disorders, and a failed suicide attempt, low socioeconomic class and a recent life stress were common findings in the histories of self-immolators.^13^ Malic et al. showed that 62.8% of the patients had a psychiatric history, 42% of the self-inflicted group had previous attempts of self-harmings. In the assault group, 12.2% of the patients were diagnosed with psychiatric disorders.^14^ While in this study, psychiatric problems were detected in 17.3% of the victims.

Familial conflicts and socioeconomic factors were the main drives leading to an unacceptably high rate of suicide by self-burning in Fars province. The problem is difficult to address and will depend precisely upon economic, educational, and social advancement for the amelioration.^11^ Analysis of the data in the current study showed familial conflict as an important risk factors for self-immolation (74.5%). Screening, identification, and education of at-risk individuals on problem solving and other aspects of coping skills, as well as interpersonal relationships, could be appropriate preventive actions and strategies to reduce self-immolation.^10^^,^^16^ Similarly, descriptive findings from Ahmadi et al. studies suggest that low family socioeconomic status was related to suicidal behavior.^16^

In order to increases socioeconomic justice, Iranian subsidy reform plan has been established by Iranian Government in December 2010 to replace subsidies on food and energy with targeted social assistance. According to Iranian targeted subsidy plan, part of the funds from energy and food subsidies will be re-allocated to the people and the remaining part will go to the industrial sectors, public education and health insurances improvement programs. This program aimed to support the social vulnerable classes and reduce the income difference between upper and lower deciles in the society,^28^ who were more susceptible to aggressive thoughts and attempts. By socioeconomic supporting of low-income and disabled families, fair distribution of the national wealth and minimizing the income disparities, the Iranian subsidy policies could reduce the incidence of self-harming behavior directly and indirectly.

Also, it is well known that availability of means to commit suicide has a major impact on actual suicidal burns in any region.^21^ The common mechanism for self-infliction of a burn is flame and using an accelerant such as petrol, which together with various paint thinners (turpentine and white spirit), rubbing alcohol or gasoline and then set fire to the propellant and clothing with a naked flame although electricity, scalding and chemicals have been used too.^13^^,^^21^^,^^29^^,^^30^

During the previous years in our country, easy accessibility to inflammable liquids such as kerosene, gasoline, mazut has decreased. According to Statistical Centre of Iran,^31^ running of natural gas line through the rural area (which consisted 57.7% of the victims in the current study) has been increased in these years, and most of rural people use natural gas instead of inflammable liquids for their domestic cooking and heating purposes. In addition, recent years an electronic-based fuel rationing program was established, in which every car had an electronic card with limited fuel quantity and if a person needed more fuel, he or she was obliged to pay more for it.

Shiraz Burn Research Center multidisciplinary preventive program against self- burning, better socioeconomic support by Iranian targeted subsidy plan**, **electronic-based fuel rationing program, widespread distribution of domestic use of natural gas instead of inflammable liquids in rural areas could be mentioned as the main reasons for promising gradual and constant decrease in incidence of self- burning in Fars Province, Iran in 2009-2012. Although the incidence of self-burning has been decreased during recent years, it is still more than western countries. Therefore, it is crucial to continue the regional preventive programs and pave the way to set up national, and even international collaborations and protocols to alleviate relevant financial, social, cultural and infrastructural difficulties to have lower incidence this terrible and dramatic issue.

## CONFLICT OF INTEREST

The authors declare no conflict of interest.
